# Cargo-interacting regions (CIR) of CCPG1 capture ER luminal cargos for reticulophagy

**DOI:** 10.1080/27694127.2023.2213560

**Published:** 2023-05-16

**Authors:** Haruka Chino, Shunsuke Ishii, Noboru Mizushima, Eisuke Itakura

**Affiliations:** a Department of Biochemistry and Molecular Biology, Graduate School of Medicine, The University of Tokyo, Tokyo, Japan; b Department of Cell Biology, Harvard Medical school, Boston, MA, USA; c Department of Biology, Graduate School of Science and Engineering, Chiba University, Chiba, Japan; d Department of Biology, Graduate school of Science, Chiba University, Chiba, Japan

**Keywords:** Aggregation, autophagy, CCPG1, ER-LAD, reticulophagy, ER-phagy

## Abstract

The endoplasmic reticulum (ER) is an organelle that regulates several vital processes necessary to maintain the health of eukaryotic cells. Protein quality control systems selectively remove abnormal ER luminal proteins to maintain ER function. In addition to the ubiquitin-proteasome pathway, the lysosome-dependent selective type of autophagy, reticulophagy (or ER-phagy), plays a crucial role in ER proteostasis. Despite the identification of several reticulophagy receptors, the mechanisms by which cytoplasmic reticulophagy machinery recognizes luminal cargo remain largely unknown. We reported that the reticulophagy receptor CCPG1 (cell cycle progression 1) contains several cargo-interacting regions (CIRs) in its ER luminal region that can directly recognize ER luminal cargos. Our findings suggest that CCPG1 is a key player in sequestering ER luminal cargo into the autophagosome using CIRs.

**Abbreviations**: CIR: cargo-interacting region; CCPG1: cell cycle progression 1; FIR: FIP200-interacting region; IAPP: islet amyloid polypeptide; LIR: LC3-interacting region;

Autophagy was originally thought to be a non-selective bulk degradation system. However, recent studies have demonstrated that some damaged or excess proteins and organelles, including mitochondria and endoplasmic reticulum (ER), are selectively degraded by autophagy. Autophagic degradation of the ER, termed reticulophagy, is a selective type of autophagy mediated by reticulophagy receptors, which link the autophagosomal membrane to the cargo. To date, eight membrane-embedded (RETREG2/FAM134A, RETREG1/FAM134B, RETREG3/FAM134C, RTN3L, CCPG1, SEC62, TEX264, and ATL3) and three soluble (CALCOCO1, CDK5RAP3/C53 and SQSTM1/p62) reticulophagy receptors have been identified in mammals. Furthermore, two membrane-embedded (Atg39 and Atg40) and a soluble (Epr1) reticulophagy receptors have been identified in yeast, such as *Saccharomyces cerevisiae* and *Schizosaccharomyces pombe*, respectively. The discovery of these receptors has increased our understanding of reticulophagy.

One of the important roles of reticulophagy is to dispose the excess ER. Deletion of reticulophagy receptors results in increased ER volume, whereas the activation of autophagy helps prevent the expansion of the ER. Another role of reticulophagy is the quality control of ER proteins. One-third of cellular proteins are synthesized in the ER and adopt their native structures with the assistance of luminal chaperones and enzymes. Increased accumulation of aberrant proteins in the ER disrupts ER homeostasis. Therefore, these abnormal proteins are eliminated by ER quality control systems. One such system is the ER-associated protein degradation (ERAD) pathway, which is mediated by the ubiquitin–proteasome system. In this pathway, misfolded proteins in the ER are retro-translocated back to the cytosol for proteasomal degradation. However, some misfolded proteins in the ER are not degraded by the ERAD but by reticulophagy, which highlights the critical role of this pathway in ER protein quality control. despite this crucial cellular role, we lack a comprehensive knowledge of the mechanisms by which cytosolic autophagy machinery selectively recognizes proteins in the ER lumen.

We recently reported that CCPG1, a reticulophagy receptor, directly recognizes ER luminal cargoes through its luminal region and targets them for autophagic degradation^1^. We hypothesized that luminal-aggregated proteins that cannot be dislocated across the ER membrane for proteasomal degradation are selectively degraded by reticulophagy. We used islet amyloid polypeptide (IAPP), whose aggregation is observed in type 2 diabetes, as a model substrate. The six-repeat IAPPs (6×IAPP) are known to form a tight oligomer. To investigate whether IAPP oligomers were degraded by reticulophagy, we generated reticulophagy reporter 6×IAPP-RFP-GFP-KDEL, consisting of 6×IAPP followed by RFP, GFP, and the ER retention signal KDEL. As RFP is more stable in lysosomes than GFP, we could monitor the lysosomal delivery of 6×IAPP. We observed that 6×IAPP localized to the ER and was degraded by reticulophagy, whereas 1×IAPP was relatively stable in the ER.

We next investigated the mechanisms by which the autophagosomal membrane recognizes 6×IAPP in the ER. We observed that only CCPG1, but not other reticulophagy receptors, interacts with 6×IAPP and that the degradation of 6×IAPP is mainly dependent on CCPG1. CCPG1 is a type II transmembrane protein upregulated during ER stress, which helps maintain proteostasis within the lumen of the rough ER in pancreatic acinar cells. Among reticulophagy receptors, only CCPG1 has a long luminal domain. The N-terminal cytosolic domain of CCPG1 interacts with RB1CC1/FIP200 and LC3/GABARAP family proteins via the FIP200-interacting regions (FIRs) and LC3-interacting region (LIR), respectively. However, the function of the luminal domain remains to be establsihed.

Using immunoprecipitation mass spectrometry (IP-MS), we identified endogenous ER luminal proteins, such as ER chaperones, as potential CCPG1-binding proteins. Among these, we focused on one ER luminal protein, SC65/P3H4, a leprecan family member involved in regulating the lysine hydroxylation of collagen. P3H4 was also degraded by reticulophagy in a CCPG1-luminal domain-dependent manner and accumulated in autophagy-deficient mouse tissues. Functional analysis of CCPG1 using CCPG1-KO cells and in vitro binding assays with CCPG1 deletion constructs revealed that the luminal domain of CCPG1 contains cargo-interacting regions (CIRs) conserved among vertebrates. CIR1 and CIR3 are required for lysosomal degradation of 6×IAPP, whereas CIR2 is required for lysosomal degradation of P3H4. Our results suggest that CCPG1 is a bispecific reticulophagy receptor for ER luminal cargoes. The CIRs of CCPG1 can simultaneously interact with multiple substrates in the ER, whereas the LIR and FIR of CCPG1 interact with FIP200 and LC3 in the cytosol during reticulophagy (Figure 1).

**Figure 1.**
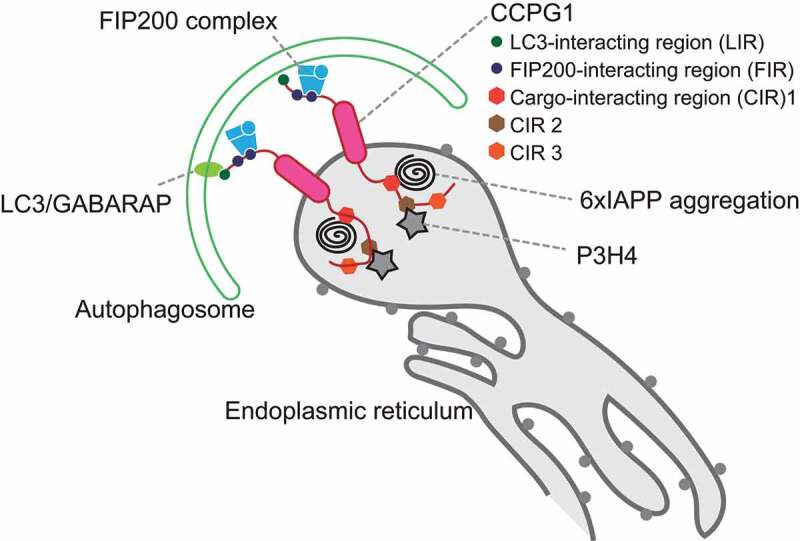
Cargo-interacting regions (CIRs) of the luminal domain of CCPG1 (cell cycle progression 1) recognize luminal cargos, such as 6×IAPP and P3H4. FIR and LIR in the cytosolic domain of CCPG1 interact with FIP200 and LC3/GABARAP family, respectively. Autophagosomes can recognize ER luminal cargos through LC3, FIP200, and CCPG1.

Our findings helped identify several aspects that warrant further investigation. First, the determinants of cargo selectivity for each CIR must be identified. We could not identify any proteins with domains similar to the CIRs using database searches. Furthermore, AlphaFold2 predicted that each one of the CIRs in CCPG1 adopts a different structures, suggesting the possibility that each CIR recognizes specific cargo in a different manner. Second, the mechanisms by which luminal cargo drive reticulophagy requires further elucidation. Since the exogenous expression of CCPG1 alone was sufficient to induce reticulophagy, ER luminal cargos may induce reticulophagy by promoting CCPG1 expression. Concordantly, CCPG1 levels were increased by the expression of 6×IAPP. However, further studies are required to elucidate the molecular mechanisms associated with CCPG1 cargo recognition and subsequent reticulophagy induction.
